# Optimal Systolic Blood Pressure for the Prevention of All-Cause and Cardiovascular Disease Mortality in Older Adults With Hypertension: Nationwide Population-Based Cohort Study

**DOI:** 10.2196/52182

**Published:** 2024-06-11

**Authors:** Jae Jun Lee, Kyung Hee Lee

**Affiliations:** 1 Mo-Im Kim Nursing Research Institute Yonsei University College of Nursing Seoul Republic of Korea

**Keywords:** aged, blood pressure, cardiovascular diseases, hypertension, mortality, older adults, geriatric, elderly, cardiovascular, Korea, Korean, insurance, cohort study, systolic, risk, aging, health outcome

## Abstract

**Background:**

Target systolic blood pressure (SBP) levels for older adults with hypertension vary across countries, leading to challenges in determining the appropriate SBP level.

**Objective:**

This study aims to identify the optimal SBP level for minimizing all-cause and cardiovascular disease (CVD) mortality in older Korean adults with hypertension.

**Methods:**

This retrospective cohort study used data from the National Health Insurance Service database. We included older adults aged 65 years or older who were newly diagnosed with hypertension and underwent a National Health Insurance Service health checkup in 2003-2004. We excluded patients who had a history of hypertension or CVD, were not prescribed medication for hypertension, had missing blood pressure or any other covariate values, and had fewer than 2 health checkups during the follow-up period until 2020. We categorized the average SBP levels into 6 categories in 10 mm Hg increments, from <120 mm Hg to ≥160 mm Hg; 130-139 mm Hg was the reference range. Cox proportional hazards models were used to examine the relationship between SBP and all-cause and CVD mortalities, and subgroup analysis was conducted by age group (65-74 years and 75 years or older).

**Results:**

A total of 68,901 older adults newly diagnosed with hypertension were included in this study. During the follow-up period, 32,588 (47.3%) participants had all-cause mortality and 4273 (6.2%) had CVD mortality. Compared to older adults with SBP within the range of 130-139 mm Hg, individuals who fell into the other SBP categories, excluding those with SBP 120-129 mm Hg, showed significantly higher all-cause and CVD mortality. Subgroup analysis showed that older adults aged 65-74 years had higher all-cause and CVD mortality rates according to SBP categories than those aged 75 years or older.

**Conclusions:**

The SBP levels within the range of 120-139 mm Hg were associated with the lowest all-cause and CVD mortality rates among older Korean adults with hypertension. It is recommended to reduce SBP to <140 mm Hg, with 120 mm Hg as the minimum value for SBP, for older Korean adults with hypertension. Additionally, stricter SBP management is required for adults aged 65-74 years.

## Introduction

The older adult population is growing globally at a faster rate than that in previous years [1]. The World Health Organization expects the number of older adults to rise from 1 to 2 billion between 2019 and 2050, and in 2050, 22% of the world’s population is expected to comprise older adults [1]. In Korea, the aging population is progressing more rapidly than in other countries; it is expected that the proportion of the older adult population will exceed 20% in 2025, 30% in 2035, and 40% in 2050 [2]. To maintain quality of life with longevity, managing chronic disease is important in this population.

Cardiovascular disease (CVD), such as myocardial infarction, stroke, and heart failure, is the leading cause of death worldwide [3], and hypertension is one of the primary modifiable risk factors for CVD mortality [4]. Hypertension is very common in older adults because their systolic blood pressure (SBP) tends to be elevated due to increased stiffness and decreased elasticity in the arteries with aging [5]. Epidemiologic research by the United States National Health and Nutrition Examination Survey has indicated that 76.5% of older adults aged older than or equal to 65 years have hypertension [6]. Moreover, older adults with hypertension are more susceptible to CVD than younger adults with hypertension [7]; therefore, controlling the blood pressure of older adults is extremely important to prevent CVD.

However, the guidelines for target blood pressure levels in older adults with hypertension are inconsistent across countries. In 2017, the American College of Cardiology and the American Heart Association (ACC/AHA) revised the guidelines for the diagnostic criteria of hypertension from 140/90 mm Hg to 130/80 mm Hg. They recommend a target SBP level of <130 mm Hg in older adults with hypertension [8]. These guidelines are equivalent to the 2022 Taiwan Hypertension Guidelines of the Taiwan Society of Cardiology and the Taiwan Hypertension Society for the management of hypertension, recommending that blood pressure treatment in older adults should be initiated when SBP is ≥130 mm Hg; the target SBP level is <130 mm Hg [9]. On the other hand, the European Society of Cardiology and the European Society of Hypertension guidelines of 2018 maintain the previous criterion of hypertension as >140/90 mm Hg and recommend a target SBP range of 130-139 mm Hg, while advising against lowering it below 130 mm Hg due to potential risks without additional benefits [10]. This is similar to the 2019 Chinese guideline for the management of hypertension in older people, which recommends a target blood pressure level of <140/90 mm Hg [11]. The differences in target SBP levels across countries are related to variability in the prevalence of hypertension and CVD mortality rates among different racial and ethnic groups or regions [6,12,13]. Therefore, the diagnostic and treatment guidelines of hypertension are different across countries, making it difficult to achieve optimal blood pressure management in older adults and leading to challenges in determining the appropriate interventions.

In Korea, the 2022 guidelines for the management of hypertension published by the Korean Society of Hypertension (KSH) still maintain the diagnostic criterion for hypertension as >140/90 mm Hg [14]. Additionally, they recommend lowering SBP to <140 mm Hg for older adults with hypertension [14]. However, the KSH guidelines have a limitation in that they are based on research findings that may not specifically include data from older adults in Korea [15-18]. Therefore, further studies are needed to explore the optimal blood pressure levels for older Korean adults with hypertension.

In addition, previous studies conducted to determine the optimal blood pressure levels for older adults with hypertension have been limited by not investigating the impact of age differences. The 2019 Japanese Society of Hypertension guidelines recommend different target blood pressures for different age categories—<130/80 mm Hg in patients aged younger than 75 years and <140/90 mm Hg in those aged 75 years and older. This is because lowering SBP to <130 mm Hg in individuals aged 75 years or older may potentially result in adverse health outcomes, such as renal dysfunction [19]. However, to the best of our knowledge, no studies have identified age differences in optimal SBP levels in older Korean adults. Analyzing the impact of age-related differences in SBP on all-cause and CVD mortalities among older Korean adults with hypertension is an important indicator of future blood pressure management. Therefore, this study aimed to investigate (1) the effects of SBP on all-cause and CVD mortalities in older adults with hypertension and (2) the associations stratified by age (65-74 years and 75 years or older).

## Methods

### Study Design and Sample

This retrospective cohort study used data from the National Health Insurance Service (NHIS) database. The NHIS is a single obligatory social insurance system covering health care service costs in Korea. The entire population residing in Korea subscribes to mandatory social insurance, and all medical care institutions claim medical fees. The claims include medical information, such as diagnosis, prescription, medication, and data collected from the NHIS database. Because the NHIS also provides general health checkups biennially for all insured adults (annually for nonoffice workers) and collects health checkup data, the NHIS database is useful for continuously observing the health status of the Korean population. The NHIS database was initiated in 2002 and we followed up data until 2020.

**Figure 1:**
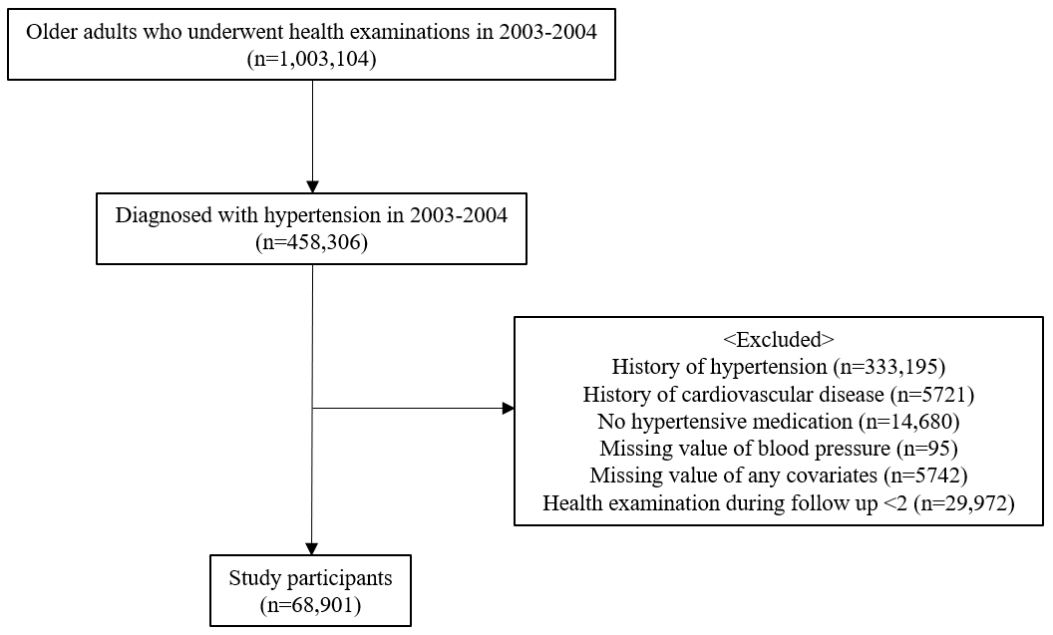
Characteristics of the study participants.

We included older adults who (1) were aged 65 years or older, (2) were newly diagnosed with hypertension (Korean Classification of Disease [KCD] code I10), and (3) underwent the NHIS health checkup in 2003-2004. We excluded patients who (1) had a history of hypertension or CVD, (2) were not prescribed medication for hypertension, (3) had missing blood pressure or any other covariate values, and (4) had fewer than 2 health checkups during the follow-up period. Characteristics of the study population are shown in Figure 1. A total of 68,901 participants were eligible and were followed up until 2020.

### Ethical Considerations

Ethics approval was obtained from the institutional review board of Yonsei University Health System (4-2021-1277), and the study complied with the Declaration of Helsinki. The NHIS database was anonymized to ensure confidentiality, and informed consent was not required because this was retrospective study using the NHIS database.

### Measurement

The independent variable in our study was the mean SBP measured during the health checkups. Blood pressure was measured using an automatic blood pressure monitor placed in front of the examiner after the participant had stabilized for at least 5 minutes. If the SBP was >120 mm Hg or the diastolic blood pressure was >80 mm Hg, the blood pressure measurement was repeated after at least 2 minutes. In this study, we categorized the SBP into 6 categories in 10 mm Hg increments from <120 mm Hg to ≥160 mm Hg, and 130-139 mm Hg was used as a reference category.

The dependent variables were all-cause and CVD mortalities. The CVD mortality was defined as death from myocardial infarction (KCD codes I21-23), heart failure (KCD code I50), hemorrhagic stroke (KCD codes I60-62), or ischemic stroke (KCD code I63). Data on the dates and causes of mortality were obtained from Statistics Korea.

Sociodemographic characteristics, health behaviors, blood test results, and hypertension medications were included as covariates. The sociodemographic characteristics included age, sex, and BMI. Age was classified as 65-74 years and 75 years or older. Health behaviors included smoking status, physical activity, and the Charlson Comorbidity Index. Blood test results included fasting glucose, total cholesterol, aspartate transaminase, and alanine transaminase. Hypertension medications included renin-angiotensin system blockers (angiotensin-converting enzyme inhibitors and angiotensin receptor blockers), beta-blockers, dihydropyridine calcium channel blockers, and diuretics.

### Statistical Analysis

Descriptive analyses were used to describe sample characteristics. Continuous variables were described as means (SDs), and categorical variables were described as numbers and percentages. Thereafter, Kaplan-Meier curves and Cox proportional hazards models were used to identify the relationship between SBP and all-cause and CVD mortalities after adjusting for other covariates. A subgroup analysis was performed to identify age-related differences in the risk of all-cause and CVD mortalities according to SBP. All statistical analyses were performed using SAS (version 9.4; SAS Institute Inc), and the significance level was set at *P*<.05.

## Results

In total, 68,901 older adults newly diagnosed with hypertension were included in this study. We divided them into the following SBP categories: 5666 (8.2%) as <120 mm Hg, 17,236 (25.0%) as 120-129 mm Hg, 25,277 (36.7%) as 130-139 mm Hg, 14,299 (20.8%) as 140-149 mm Hg, 4663 (6.8%) as 150-159 mm Hg, and 1760 (2.6%) as ≥160 mm Hg. Among the participants, 57,831 (83.9%) were aged 65-74 years and 11,070 (16.1%) were aged 75 years or older. The general characteristics of the study participants are presented in Table 1.

**Figure 2:**
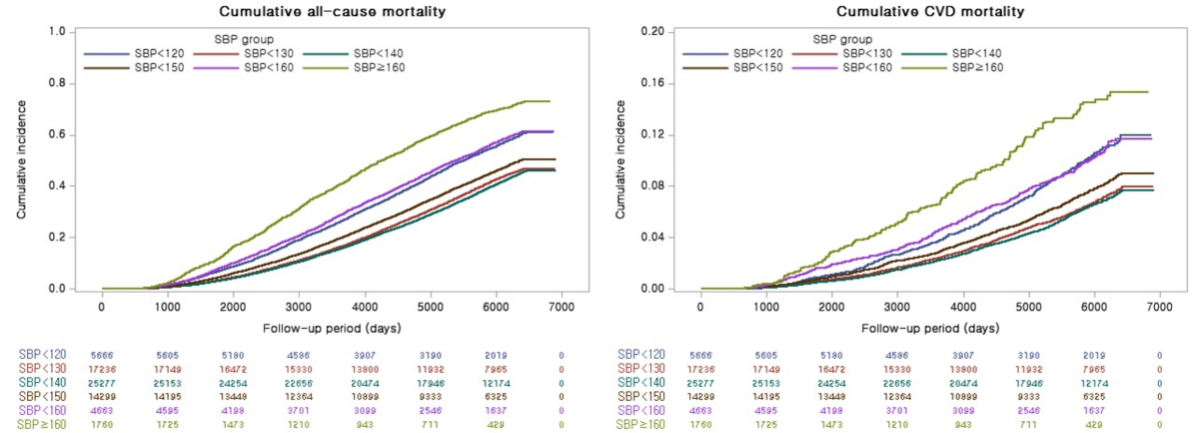
Kaplan-Meier curves for all-cause and CVD mortality according to SBP. CVD: cardiovascular disease; SBP: systolic blood pressure.

Figure 2 shows the Kaplan-Meier curves for the cumulative incidence of all-cause and CVD mortalities according to the SBP category. The incidence of all-cause and CVD mortalities varied between SBP categories; older adults with SBP 160 mm Hg had the highest mortality rate, while those with SBP within the range of 130-139 mm Hg had the lowest mortality rate.

**Table 1:** General characteristics of the study participants (N=68,901).

Variables			Values
**Age (years), n (%)**			
	65-74	57,831 (83.9)	
	≥75	11,070 (16.1)	
**Sex, n (%)**			
	Male	30,995 (45)	
	Female	37,906 (55)	
**BMI, n (%)**			
	Underweight (<18.5 kg/m^2^)	2175 (3.2)	
	Normal weight (<23 kg/m^2^)	23,413 (34)	
	Overweight (<25 kg/m^2^)	18,240 (26.5)	
	Obese (≥25 kg/m^2^)	25,073 (36.4)	
**Smoking status, n (%)**			
	Nonsmoking	53,231 (77.3)	
	Quit smoking	5672 (8.2)	
	Current smoking	9998 (14.5)	
**Physical activity, n (%)**			
	None	47,038 (68.3)	
	1-2 times per week	9324 (13.5)	
	3 or more times per week	12,539 (18.2)	
**CCI^a^, n (%)**			
	0	34,443 (50)	
	1-2	20,027 (29.1)	
	3 or more	14,431 (20.9)	
Fasting glucose, mean (SD)			103.6 (38.6)
Total cholesterol, mean (SD)			204.9 (66.3)
AST^b^, mean (SD)			27.4 (18.2)
ALT^c^, mean (SD)			23.3 (19.2)
**Medication, n (%)**			
	RASB^d^	24,719 (35.9)	
	BB^e^	7116 (10.3)	
	CCB^f^	35,902 (52.1)	
	Diuretics	38,703 (56.2)	

^a^CCI: Charlson Comorbidity Index.

^b^AST: aspartate transaminase.

^c^ALT: alanine transaminase.

^d^RASB: renin-angiotensin system blocker.

^e^BB: β-blocker.

^f^CCB: dihydropyridine calcium channel blocker.

Table 2 shows the results of the Cox proportional hazards models for all-cause and CVD mortalities according to SBP categories. During the follow-up period, 32,588 (47.3%) patients had all-cause mortality and 4273 (6.2%) had CVD mortality. Compared to participants with SBP within the range of 130-139 mm Hg, those with SBP <120 mm Hg had a significantly higher all-cause (hazard ratio [HR] 1.40, 95% CI 1.35-1.46) and CVD mortality (HR 1.42, 95% CI 1.27-1.58) rate. These results were similar in participants with SBP within the range of 140-149 mm Hg, 150-159 mm Hg, and ≥160 mm Hg, in that the participants falling into these SBP categories had significantly higher all-cause and CVD mortality rates compared with those with SBP within the range of 130-139 mm Hg. However, when compared with participants with SBP within the range of 130-139 mm Hg, those with SBP within the range of 120-129 mm Hg did not show a significant difference in either all-cause (HR 1.02, 95% CI 0.99-1.05) or CVD mortality (HR 1.00, 95% CI 0.92-1.09).

**Figure 3:**
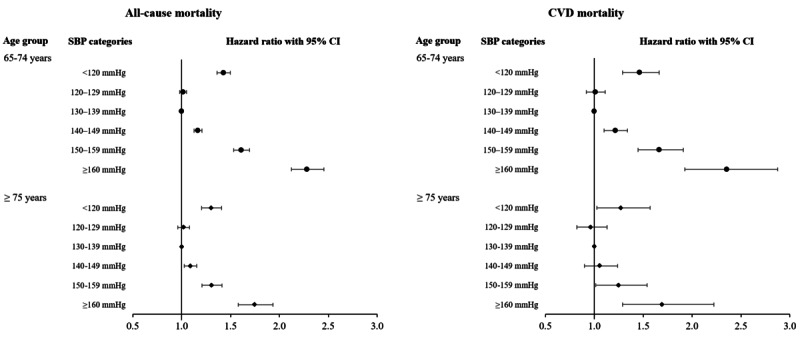
Subgroup analysis of the effect of SBP on all-cause and CVD mortality stratified by the age groups of 65-74 years and 75 years or older. CVD: cardiovascular disease; SBP: systolic blood pressure.

Results of the subgroup analysis for the effect of SBP on mortality stratified by age (65-74 years and 75 years or older) are shown in Figure 3. In both age categories, there was a significantly lower mortality rate in the 130-139 mm Hg SBP category than in the other SBP categories, except for the 120-129 mm Hg category. Moreover, those aged 65-74 years showed a higher HR of all-cause and CVD mortalities according to the SBP categories than those aged 75 years or older.

**Table 2:** Cox proportional hazards models^a^ to predict all-cause and CVD^b^ mortalities by SBP^c^.

Events and SBP categories (mm Hg)		Number of events, n (%)	HR^d^ (95% CI)
**All-cause mortality**			
	<120	3262 (57.6)	1.40 (1.35-1.46)
	120-129	7641 (44.3)	1.02 (0.99-1.05)
	130-139	10,833 (42.9)	1^e^
	140-149	6862 (48.0)	1.15 (1.12-1.19)
	150-159	2745 (58.9)	1.51 (1.45-1.58)
	≥160	1245 (70.7)	2.08 (1.96-2.21)
**CVD mortality**			
	<120	437 (7.7)	1.42 (1.27-1.58)
	120-129	984 (5.7)	1 (0.92-1.09)
	130-139	1408 (5.6)	1^e^
	140-149	912 (6.4)	1.17 (1.08-1.28)
	150-159	364 (7.8)	1.53 (1.36-1.71)
	≥160	168 (9.5)	2.11 (1.80-2.48)

^a^Statistical analysis adjusted for age, sex, BMI, smoking status, physical activity, Charlson Comorbidity Index, fasting glucose, total cholesterol, AST, ALT, and blood pressure medication.

^b^CVD: cardiovascular disease.

^c^SBP: systolic blood pressure.

^d^HR: hazard ratio.

^e^Reference value.

## Discussions

### Principal Findings

In this retrospective cohort study, the rates of all-cause and CVD mortalities were significantly lower in older adults with hypertension and SBP within the range of 130-139 mm Hg than in the other SBP categories, except for those with SBP within the range of 120-129 mm Hg. These findings suggest the importance of managing SBP within the range of 120-139 mm Hg to lower all-cause and CVD mortality risks in older adults with hypertension. The optimal target SBP level was 120-139 mm Hg in both age groups (65-74 years and 75 years of age or older), and the impact of SBP on mortality was generally higher in older adults aged 65-74 years, indicating that more stringent SBP control is required in these patients than in their older counterparts.

Our results are consistent with the SBP goal of the 2022 KSH guidelines of <140 mm Hg [14]. The 2022 KSH guidelines have limitations in that the target SBP level for older adults with hypertension was established based on the findings of studies conducted on non-Korean participants [15-18]. Additionally, 1 of the main reasons for the unchanged target SBP level of the 2022 KSH guidelines compared to the 2018 KSH guidelines is that there is still insufficient evidence regarding the optimal target SBP level [14]. Our study provides empirical evidence for the optimal SBP level of older adults with hypertension in Korea to reduce all-cause and CVD mortality rates.

This study showed the presence of a J-curved relationship between SBP and CVD risk, indicating that lowering SBP to below 120 mm Hg increased all-cause and CVD mortalities compared with SBP within the range of 130-139 mm Hg. This result was consistent with an international cohort study on 22,672 patients with coronary artery disease [20] and the data from 2 randomized controlled trials (RCTs) on 30,937 patients with high cardiovascular risk [21], which showed that SBP within the range of 120-139 mm Hg had the lowest risk of cardiovascular events, and the risk increased at SBP <120 mm Hg, with a J-curve pattern. Furthermore, 19,110 older adults aged older than or equal to 60 years in the Singapore Chinese Health Study showed that SBP <120 mm Hg tended to increase CVD mortality risk compared with SBP between 120-139 mm Hg, although the risk estimates did not reach statistical significance [22]. However, most guidelines for hypertension management, including the ACC/AHA and KSH guidelines, do not specify the minimal level of SBP. It may be appropriate to suggest the minimum SBP level when scientists and professionals update the hypertension management guidelines.

Although several studies on the optimal target SBP level have been conducted, the results remain controversial across countries. The Systolic Blood Pressure Intervention Trial (SPRINT) in the United States showed that an SBP target of <120 mm Hg decreased major adverse cardiovascular events and mortality compared to an SBP target of <140 mm Hg in older adults with hypertension aged 75 years or older [15]. The Strategy of Blood Pressure Intervention in the Elderly Hypertensive Patients trial in China showed that an SBP target of 110-130 mm Hg significantly reduced major adverse cardiovascular events compared to an SBP target of 130-150 mm Hg [16]. The Berlin Initiative Study in Germany showed that SBP of <130 mm Hg is associated with increased all-cause mortality compared to SBP within the range of 140-149 mm Hg [23], whereas another cohort study in England showed that 6311 individuals aged 50-79 years and 1299 individuals aged 80-89 years had no significant associations between SBP levels and all-cause and CVD mortalities [24]. A possible explanation for the different results is ethnic disparities. The prevalence of hypertension and CVD mortality differs across countries [6,12,13]. Asian populations are known to be more vulnerable to hypertension because they tend to have an increased aortic pulse and stiffness than European populations [25], and Asians have a stronger association between blood pressure and CVD than Europeans [25]. Therefore, the target SBP level for hypertension may vary according to race and country.

The estimated risk for all-cause and CVD mortalities increased for every 10 mm Hg increase in SBP above 140 mm Hg, compared to within the 130-139 mm Hg range, regardless of age group, although the impact of SBP on mortality was slightly higher in individuals aged 65-74 years. However, the relationship between SBP and mortality in older adults remains controversial. A total of 2 RCTs, involving patients aged 75 years and older in the SPRINT [15] and 80 years or older in the Hypertension in the Very Elderly Trial [26], showed that a lower SBP was associated with decreased cardiovascular events. However, several cohort studies suggested that there was no association in older adults 75 years or older [27,28], and some even showed a negative relationship between SBP and cardiovascular events [29,30]. The discrepancies between the RCTs and cohort studies might be that the participants of RCTs were healthier than the general population of the same age because of the restricted inclusion and exclusion criteria [31]. The reason our study revealed an association between increased SBP and higher mortality, unlike other cohort studies, could be attributed to our deliberate selection of healthy older adults who were capable of undergoing health checkups on their own and had no previous history of hypertension or CVD. Therefore, further studies targeting diverse populations (ie, frail or multimorbid) are needed.

### Limitations and Strengths

This study had several limitations. Because we included participants who underwent health checkups between 2003 and 2004, older adults who did not undergo health checkups were excluded. We adjusted for many covariates to alleviate bias, but potential age-related confounding variables, such as frailty, activities of daily living, and gait speed, remained due to data availability. In addition, adverse events of decreased blood pressure, such as renal dysfunction or orthostatic hypotension, were not considered in this study due to data limitations; therefore, we suggest that additional studies exploring optimal blood pressure should include adverse events as 1 of the outcomes. Finally, we used a conventional Cox hazard model with mean SBP measured during the health checkups as an independent variable to facilitate interpretation. However, since SBP is a time-varying variable, a time-dependent Cox model could also have been used. The strengths of this study included the use of the NHIS database, which is nationally representative and includes almost all Koreans, and a large sample size. Our results have sufficient statistical power and can be generalized to older Korean adults with hypertension. In addition, through a 17-year follow-up observation period, our study provided evidence that controlling SBP is crucial for long-term CVD-related health outcomes.

### Conclusions

Controlling SBP in older adults with hypertension is important for improving overall and CVD-related health outcomes. However, hypertension guidelines regarding optimal SBP levels differ across countries; therefore, it is necessary to identify the appropriate SBP level among older Korean adults with hypertension. Our retrospective cohort study using national data showed that SBP within the range of 120-139 mm Hg was associated with the lowest risk of CVD and all-cause mortality in older Korean adults with hypertension; therefore, SBP should be controlled within this range in this cohort. Our study also suggested that lowering the SBP to below 120 mm Hg could increase all-cause and CVD mortalities, indicating that caution should be taken not to lower the SBP beyond 120 mm Hg. Additionally, SBP had a greater impact on CVD and all-cause mortality in older adults aged 65-74 years than in those aged 75 years and older; more stringent SBP management is thus required in the 65-74-year age group. This study provides critical knowledge that improves our understanding of the optimal SBP in older Korean adults with hypertension to reduce all-cause and CVD mortality rates. The KSH guidelines only recommend lowering SBP to <140 mm Hg; however, we suggest adding 120 mm Hg as the minimum value.

## Abbreviations

ACC/AHA: American College of Cardiology and the American Heart Association
CVD: cardiovascular disease
HR: hazard ratio
KCD: Korean Classification of Disease
KSH: Korean Society of Hypertension
NHIS: National Health Insurance Service
RCT: randomized controlled trial
SBP: systolic blood pressure
SPRINT: Systolic Blood Pressure Intervention Trial
